# Syngnathoid Evolutionary History and the Conundrum of Fossil Misplacement

**DOI:** 10.1093/iob/obad011

**Published:** 2023-05-04

**Authors:** C D Brownstein

**Affiliations:** Department of Ecology and Evolutionary Biology, Yale University, New Haven CT, USA; Stamford Museum and Nature Center, 359 Merriebrook Lane Stamford CT, 06902, USA

## Abstract

Seahorses, pipefishes, trumpetfishes, shrimpfishes, and allies are a speciose, globally distributed clade of fishes that have evolved a large number of unusual body plans. The clade that includes all these forms, Syngnathoidei, has become a model for the study of life history evolution, population biology, and biogeography. Yet, the timeline of syngnathoid evolution has remained highly contentious. This debate is largely attributable to the nature of the syngnathoid fossil record, which is both poorly described and patchy for several major lineages. Although fossil syngnathoids have been used to calibrate molecular phylogenies, the interrelationships of extinct species and their affinities to major living syngnathoid clades have scarcely been quantitatively tested. Here, I use an expanded morphological dataset to reconstruct the evolutionary relationships and clade ages of fossil and extant syngnathoids. Phylogenies generated using different analytical methodologies are largely congruent with molecular phylogenetic trees of Syngnathoidei but consistently find novel placements for several key taxa used as fossil calibrators in phylogenomic studies. Tip-dating of the syngnathoid phylogeny finds a timeline for their evolution that differs slightly from the one inferred using molecular trees but is generally congruent with a post-Cretaceous diversification event. These results emphasize the importance of quantitatively testing the relationships of fossil species, particularly when they are critical to assessing divergence times.

## Introduction

Phylogenies of species-rich clades have proliferated as genomic sequencing techniques have rapidly progressed, positioning phylogenetics at the center of studies of evolution (Smith et al. 2020). Although early phylogenetic analyses of extant clades used morphological datasets and often included fossils, the role of extinct species in reconstructing the tree of life is being reevaluated. Particularly, fossils are known to drastically change topologies and divergence times in morphological and combined evidence analyses where fossils are included as terminal taxa (Pyron 2011; Ronquist et al. 2012; Arcila et al. 2015; Gavryushkina et al. 2017; Lee and Yates 2018; Luo et al. 2020; Mongiardino Koch and Parry 2020; Simões et al. 2020; Mongiardino Koch et al. 2021; Mongiardino Koch and Thompson 2021; Cole et al. 2022). The proliferation of studies using fossils to date phylogenies as either node or tip calibrators has established the need for comprehensive analyses of morphological data to determine the placement of extinct species among living clades (Parham et al. 2012).

Syngnathoidei is a global radiation of teleost fishes that has produced several unusual body plans and life history traits, including dermal armor and exoskeletons (Orr 1995; Praet et al. 2010; Stiller et al. 2015), male pregnancy (Wilson et al. 2001; Wilson et al. 2003; Wilson and Orr 2011; Roth et al. 2020), extensive camouflage that includes leaf-like appendages (Stiller et al. 2015; Qu et al. 2021; Small et al. 2022), upright posture (Teske and Beheregaray 2009), and the loss of pelvic and caudal fins (Lin et al. 2016). Given the apparently high evolvability of the phenotype in Syngnathoidei, the evolutionary relationships of fishes in this clade have been heavily studied using molecular sequence data (e.g., Teske and Beheregaray 2009; Wilson and Rouse 2010; Hamilton et al. 2017; Longo et al. 2017; Li et al. 2021; Qu et al. 2021; Santaquiteria et al. 2021; Stiller et al. 2022). Although the most recent explorations of syngnathoid phylogeny have used the rapidly growing fossil record of this clade to node-calibrate trees (e.g., Santaquiteria et al. 2021; Stiller et al. 2022), the integration of fossils into analyses of phenotypic evolution in seahorses and their relatives is limited (Orr 1995; Bannikov and Carnevale 2012; Cantalice and Alvarado-Ortega 2016), as are phylogenetic analyses of extinct syngnathoid species (Cantalice and Alvarado-Ortega 2016; Murray 2022). In particular, convergence among phylogenetically distant lineages in Syngnathoidei (see Stiller et al. 2022) suggests that the nearly 100-million-year (Near et al. 2013; Hughes et al. 2018; Alfaro et al. 2018; Santaquiteria et al. 2021; Ghezelayagh et al. 2022; Stiller et al. 2022) evolutionary history of this clade may have seen the emergence of extinct clades that converged on the phenotypes of major living pipefish, seahorse, trumpetfish, and shrimpfish lineages.

Here, I present a hypothesis of fossil and extant syngnathoid relationships using the most taxon- and character-rich morphological dataset constructed for this clade. Intensive sampling of fossil and extant species suggests that the supposed affinities of several early fossil species to the major extant syngnathoid clades are not supported, necessitating a reevaluation of fossil calibrators of this lineage.

## Methods

### Nomenclatural note

I use the prefix pan- to refer to the total clade of a lineage (i.e., all fossil and extant representatives, including fossils along the stem of an extant lineage that fall outside the most recent common ancestor of living species).

### Morphological dataset construction

I constructed a new morphological dataset to test the interrelationships of syngnathoid fishes using the matrix of Cantalice and Alvarado-Ortega (2016), as updated by Murray (2022). I added nine additional characters related to the phylogenetic relationships of true seahorses (*Hippocampus* spp.), pygmy pipefishes, ghost pipefishes, and syngnathin and nerophin pipefishes identified in previous studies (Teske and Beheregaray 2009; Stiller et al. 2015; Bannikov and Carnevale 2017; Neutens et al. 2017; Short et al. 2020; Short and Trnski 2021). Next, I checked and corrected for mismatches between character state descriptions and codings provided in the literature. The resulting set of morphological characters, along with notes about character state distributions, is included in the Supplement to this article.

I added seven extant species based on personal observation of YPM specimens, publicly available computed-tomography scans (Cauter et al. 2010; Lim et al. 2015; Stiller et al. 2015; Short et al. 2018; Short and Trnski 2021), and previously published anatomical studies (e.g., Orr 1995) to sample the diversity of extant syngnathids more fully (see Stiller et al. 2022). I also coded 17 additional fossil taxa for the matrix based on personal observation of specimens and the published literature (Blot 1980; Orr 1995; Žalohar et al. 2009; Přikryl et al. 2011; Bannikov and Carnevale, 2012, 2017; Žalohar and Hitij, 2012, 2017; Bannikov et al. 2017). The final matrix consisted of 36 taxa scored for 101 morphological characters, which are sampling increases of 200 and 9%, respectively, from the previous version of the dataset (Murray 2022). A table including all specimens personally examined and coded based on the literature, along with citations, is included in the Supplementary Information as Table S1.

### Parsimony analysis of the morphological dataset

Parsimony analysis in the program TNT v. 1.5 (Goloboff and Catalano 2016) started with an initial Wagner search over 10 replicates with space for 1000 trees and default parameters for ratchet, tree fuse, tree drift, and sectorial search, followed by a round of traditional bisection-reconnection branch swapping with space for 100,000 trees to further explore topological space. Aulorhynchidae was set as the outgroup. The resulting most parsimonious trees were then summarized in a strict consensus topology. Finally, bootstrap supports were generated using resampling over 100 replicates. I reran the phylogenetic analysis in TNT v. 1.5 using the same methods described above with the addition of forced constraints on the relationships of extant syngnathoids following the phylogenies presented in Santaquiteria et al. (2021) and Stiller et al. (2022) to test how the placement of fossils changed based on character optimization changes resulting from a topology that subscribed to the phylogenomic hypothesis. The most parsimonious trees recovered, the strict consensus tree, the combinable components tree with bootstrap supports, and list of resolved apomorphies for the strict consensus topology are included in the Supplementary Information.

### Bayesian analysis of the morphological dataset

Bayesian analyses of the updated morphological dataset were conducted in BEAST v. 2.6.6 (Bouckaert et al. 2019) using age dates from published calibration justifications in the literature, as the fossil taxa included in this study have been used in previous phylogenomic analyses or are found in the same assemblages as species that have been used as fossil calibrators (Santaquiteria et al. 2021; Stiller et al. 2022). I constructed an input XML file for BEAST v. 2.6.6 in the terminal BEAUTi v. 2.6.6. Character evolution was modeled using the Markov-variable (Mkv) scheme of Lewis (2001) and with partitions according to state numbers; the analysis included 99 two-state and 7 three-state characters. I employed the Fossilized Birth–Death model as implemented in BEAST2 (Gavryushkina et al. 2017) with an uncorrelated lognormal clock with default values (Drummond and Rambaut 2007). An input age for the origin was given as 100.0 million years ago (upper value = 120.0 million years ago, lower value = 90.0 million years ago), which is slightly older than the oldest definite fossil representatives of the Syngnathiformes (Orr 1995; Stiller et al. 2022) and matches the approximate divergence time of this lineage from larger phylogenomic studies (e.g., Alfaro et al. 2018; Ghezelayagh et al. 2022). The rho parameter was set to 0.46, which is the proportion of extant OTUs in the dataset. Aulorhynchidae and Scombriformes (Trichiuridae + Scombridae) were forced as successive outgroups using MRCA monophyletic constraint priors. I conducted two independent runs of the analysis using the Markov Chain Monte Carlo (MCMC) process over 1.0 × 10^8^ generations with a 1.0 × 10^7^ pre-burnin, with trees sampled every 2000 generations. Convergence of the posteriors was assessed using Tracer 1.7.1 (Rambaut et al. 2018) based on effective sample size (ESS) values and inspection of MCMC generation versus posterior value plots. The resulting posterior tree sets from the two independent runs were combined with 10% burnin each and summarized in a maximum clade credibility tree with median node heights using the tools LogCombiner v. 2.6.6 and TreeAnnotator v. 2.6.4. (Bouckaert et al. 2019). As in the analyses conducted under parsimony, I tested how enforcing the hypothesis of syngnathine interrelationships (Syngnathini as sister to Hippocampini) found using genome-scale datasets (Santaquiteria et al. 2021; Stiller et al. 2022) modified the placement of fossil taxa by using monophyletic MRCA constraint priors. All XML files and output maximum clade credibility tree files are in the Supplementary Information.

## Results

### Relationships of extinct and living syngnathoids

**Fig. 1:**
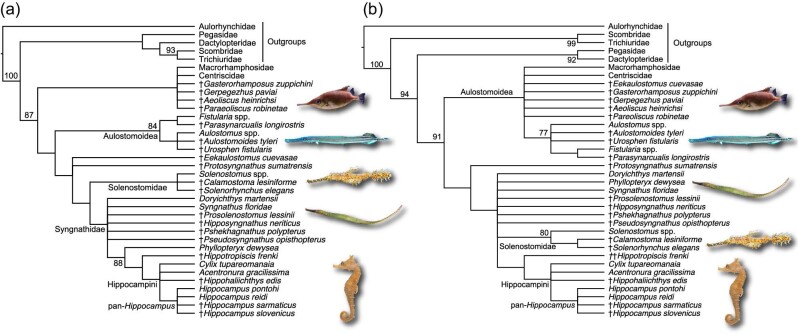
Phylogenetic hypotheses of Syngnathoidei based on parsimony analysis. Strict consensus topologies are based on (**A**) the unconstrained analysis and (**B**) the analysis where the relationships of living species are forced using constraints made in accordance with phylogenomic trees. Numbers are bootstrap values. All photographs are public domain from Wikipedia Commons.

Parsimony analysis of the new morphological dataset without constraints based on phylogenomic trees (Fig. 1A) recovered a monophyletic pan-Syngnathidae, including several putative pan-syngnathids from the Eocene of Monte Bolca, Italy (Blot 1980; Bannikov and Carnevale 2017). The clades Aulostomidae + Fistulariidae and Centriscidae + Macrorhamphosidae are found to be progressive outgroups to pan-Syngnathidae, which differs from phylogenomic studies (e.g., Santaquiteria et al. 2021; Stiller et al. 2022) and previous analyses of the morphological dataset (Cantalice and Alvarado-Ortega 2016; Murray 2022) that place both of these clades together in the superfamily Aulostomoidea. This is almost certainly due to the morphological similarities of trumpetfishes to syngnathoids, which include elongation of the body and simultaneous elongation of the rostrum but not the mouth itself, being resolved as synapomorphies of an exclusive group due to the more extensive sampling of early syngnathoids in this paper. The extinct species †*Gasterorhamphosus zuppichini* from the Late Cretaceous of Nardo, Italy (Orr 1995; Santaquiteria et al. 2021; Stiller et al. 2022) and †*Gerpegezhus paviai* from the Eocene of Russia (Bannikov and Carnevale 2017) are found to be members of the centriscid and macrorhamphosid total clade, whereas the Monte Bolca species †*Parasynarcualis longirostris* is found to be a pan-fistulariid and the Monte Bolca species †*Urosphen fistularis* and †*Aulostomoides tyleri* are found to be pan-aulostomids. All of these relationships are supported by weak bootstrap values. In contrast to previous studies (Cantalice and Alvarado-Ortega 2016; Murray 2022), the Danian Mexican species †*Eekaulostomus cuevasae* is found to be the earliest-diverging member of the clade that includes Syngnathidae and Solenostomidae (=Syngnathoidea). This result is important because †*Eekaulostomus cuevasae* has been extensively used as a fossil calibration for the aulostomoid total clade, which includes aulostomids, centriscids, fistulariids, and macrorhamphosids (Cantalice and Alvarado-Ortega 2016; Santaquiteria et al. 2021; Stiller et al. 2022). †*Protosyngnathus sumatrensis* is found to be one step closer to the solenostomid and syngnathid crown groups, closely matching its position in the phylogeny presented by Murray (2022). The relationships of a handful of fossil pipefishes, including the Eocene Monte Bolca species †*Prosolenostomus lessinii* and †*Pseudosyngnathus opisthopterus*, remain unresolved. However, †*Hippotropiscis frenki* from the Miocene of Slovenia is recovered as a pan-hippocampine, and the two Miocene pan-*Hippocampus* species from Slovenia are confirmed to be members of the true seahorse total group. Parsimony analysis with constraints on the relationships of extant species enforced based on phylogenomic analyses (Santaquiteria et al. 2021; Stiller et al. 2022) produced a strict consensus topology (Fig. 1B) that is largely similar to the tree recovered from the unconstrained topology, with the exception of †*Eekaulostomus cuevasae* falling within a polytomy of different aulostomoids.

**Fig. 2:**
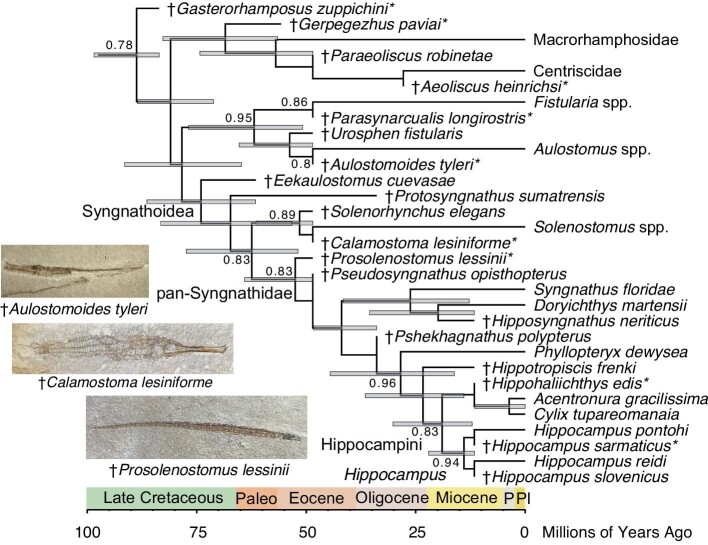
Unconstrained Bayesian time-calibrated phylogeny of Syngnathoidei. Time-calibrated maximum clade credibility tree of syngnathoid interrelationships with median node times found using BEAST 2.6.6. Numbers at nodes are posterior support values. Gray bars indicate 95% HPD divergence time intervals.

Bayesian tip-dating analysis of the dataset without constraints enforced on the syngnathoid crown group recovered a similar topology to the unconstrained parsimony analysis (Fig. 2), albeit with better resolution and higher support for particular fossils as total group members of extant clades. These include †*Parasynarcualis longirostris*, †*Aulostomoides tyleri*, and Monte Bolca solenostomids, which are all placed as total-group members of the Fistulariidae, Aulostomidae, and Solenostomidae with high (>0.85) posterior support values. The shrimpfish †*Aeoliscus heinrichsi* from the Oligocene of Germany was placed within the crown group of Centriscidae and Macrorhamphosidae as sister to the former. Syngnathidae and Solenostomidae, and the clade formed by Fistulariidae and Aulostomidae, are also supported by high posterior values, strongly suggesting †*Eekaulostomus cuevasae* is not within any major extant syngnathoid crown clade. The Monte Bolca species †*Prosolenostomus lessinii* and †*Pseudosyngnathus opisthopterus* form successive sister taxa to crown Syngnathinae, confirming their position as stem pipefishes. There is also strong support for the Miocene Slovenian true seahorses falling within the *Hippocampus* crown. Finally, †*Hipposyngnathus neriticus* is found to be the sister taxon to the single nerophin included in the dataset, and †*Hippohaliichthys edis* is recovered as a fossil relative of pygmy pipehorses; both of these relationships are supported by low posterior values.

The Bayesian maximum clade credibility tree with constraints enforced resembled the unconstrained Bayesian and parsimony trees in placing †*Eekaulostomus cuevasae* closer to Syngnathidae and Solenostomidae than to Aulostomidae and Fistulariidae. In contrast to the unconstrained Bayesian phylogeny, †*Gasterorhamposus zuppichini* is recovered as a member of the pan-Aulostomoidea rather than as the sister to crown Syngnathoidei.

### Divergence time estimation

Although the parsimony analysis conducted cannot directly infer divergence times, the placement of fossil species can set minimum bounds on the ages of key clades. For example, both unconstrained and constrained parsimony analyses imply true seahorses and the Hippocampini appeared during or before the mid-Miocene. The origins of pan-Syngnathidae, pan-Solenostomidae, pan-Aulostomidae, and pan-Fistulariidae are pushed back to the early–middle Eocene due to the position of the Monte Bolca fossils as total-group members of these clades, and the syngnathoid crown group is pushed into the Late Cretaceous due to the position of the Santonian-Campanian (see possible conflicting ages in Santaquiteria et al. 2021; Stiller et al. 2022) species †*Gasterorhamposus zuppichini* within or sister to the pan-Aulostomoidea.

**Fig. 3:**
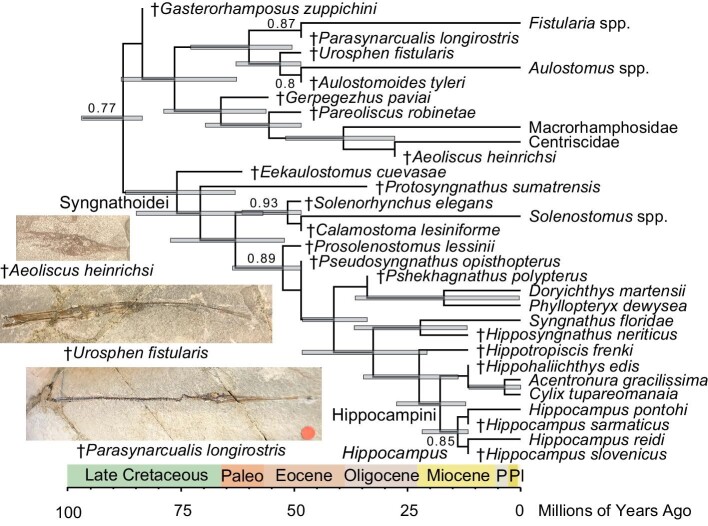
Constrained Bayesian time-calibrated phylogeny of Syngnathoidei. Time-calibrated maximum clade credibility tree of syngnathoid interrelationships with median node divergence times and constraints enforced on living species interrelationships based on phylogenomic trees found using BEAST 2.6.6. Numbers at nodes are posterior support values. Gray bars indicate 95% HPD divergence time intervals.

Divergence time estimates all had wide confidence intervals in both the unconstrained and constrained Bayesian maximum clade credibility trees (Figs. 2 and 3). Nonetheless, the divergence times estimated in this study were all broadly congruent with previous estimates based on phylogenomic data calibrated using fossils, affirming the importance of fossil data for strong divergence time inference (Table 1). In the unconstrained analysis, pan-Syngnathoidei was estimated to diverge at 88.75 Ma (95% highest posterior density [HPD] interval: 83.6–98.47 Ma), placing the origins of this clade and its closest relatives, the Pegasidae, Dactylopteridae, Callionymoidei, and Mullidae, into the early Late Cretaceous. Crown syngnathoids were estimated to diverge at 80.99 Ma (95% HPD: 71.19–88.55 Ma), and the syngnathid total group was estimated to originate at 78.38 Ma (95% HPD: 64.79–91.46 Ma). In contrast, the crown clades formed by (1) centriscids and macrorhamphosids, (2) aulostomids and fistulariids, and (3) syngnathids and solenostomids were all found to originate in the early to middle Paleocene, with median node ages of 56.96 Ma (95% HPD: 48.5–74.3 Ma), 62.88 Ma (95% HPD: 50.8–76.85 Ma), and 63.52 Ma (95% HPD: 51.87–77.41 Ma), respectively.

**Table 1:** Divergence times of major syngnathoid clades estimated in this study compared with previous studies.

Clade	Median age (Ma)	95% highest posterior density interval (Ma)	Study
Syngnathoidei	87.86	83.6–97.04	This paper with constraint
	83.09	79.07–89.75	Santaquiteria et al. (2021)
	85.51	Not given	Stiller et al. (2022)
Syngnathidae^a^	52.55	48.5–63.65	This paper with constraint
	63.1	57.5–69.5	Santaquiteria et al. (2021)
	58.29	49.90–67.11	Stiller et al. (2022)
*Hippocampus*	13.9	11.6–21.76	This paper with constraint
	14	11–16	Santaquiteria et al. (2021)
	20.8	15.97–25.86	Stiller et al. (2022)

^a^The tree presented in this study does not include the earliest diverging syngnathid clades as found in Santaquiteria et al. (2021) and Stiller et al. (2022). As such, pan-Syngnathidae, which is calibrated by the same fossils used to calibrate the branch underlying the crown node in those studies, is given.

In contrast, the ages of these three crown clades in the maximum clade credibility tree, where the relationships of extant species were constrained based on phylogenomic studies, were found to be consistently younger. The divergence at the base of crown syngnathoids was estimated to occur at 87.86 Ma (95% HPD: 83.6–97.04 Ma), and the divergence of crown Aulostomoidea was found to occur at 76.47 Ma (95% HPD: 62.77–88.28 Ma). Solenostomidae and Syngnathidae were estimated to split 63.04 Ma (95% HPD: 52.21–77.37 Ma), Fistulariidae and Aulostomidae were estimated to split 60.01 Ma (95% HPD: 50.53–72.89 Ma), and Centriscidae and Macrorhamphosidae were estimated to last share common ancestry at 39.15 Ma (95% HPD: 27.82–51.91 Ma). Hippocampini and Syngnathini were found to split 32.58 Ma (95% HPD: 20.67–48.29 Ma), and *Hippocampus* was found to split from pygmy pipehorses at 17.79 Ma (95% HPD: 12.14–27.42 Ma).

## Discussion

### Reevaluating the affinities of fossil syngnathoids: implications for phenotypic evolution

In this study, I have presented a new hypothesis of the relationships of fossil syngnathoids (seahorses, pipefishes, seadragons, ghost pipefishes, trumpetfishes, shrimpfishes, and snipefishes) to the major extant crown clades. Several species, including the Late Cretaceous taxon †*Gasterorhamposus zuppichini* and the Danian †*Eekaulostomus cuevasae*, are found to have novel placements among Syngnathoidei that differ from previously hypothesized relationships for these species (Orr 1995; Cantalice and Alvarado-Ortega 2016; Murray 2022).

The recovery of these novel relationships prompts a reevaluation of the tempo and mode of syngnathoid morphological diversification. First, the position of †*Gasterorhamposus zuppichini* as either a stem member of Centriscidae + Macrorhamphosidae or as a pan-syngnathoid confirms that the initial divergences in Syngnathoidei and its larger parent clade, the Syngnathiformes, took place in the Cretaceous. †*Gasterorhamposus zuppichini* is one of the oldest members of the percomorph crown group, which includes a large percentage of extant ray-finned fishes, and is a key calibration point for acanthomorph phylogenies (e.g., Near et al. 2013; Eytan et al. 2015; Alfaro et al. 2018; Dornburg and Near, 2021; Ghezelayagh et al. 2022) and studies of the Syngnathiformes (Santaquiteria et al. 2021; Stiller et al. 2022). Despite this, the phylogenetic position of †*Gasterorhamposus zuppichini* has never been comprehensively tested, meaning that the use of this taxon as a calibration was based solely on preliminary anatomical comparisons (Orr 1995). By confirming that †*Gasterorhamposus zuppichini* falls outside any of the major crown clades in Syngnathoidei, this study provides hard evidence for justifying the use of this pivotal taxon as a fossil calibration point. Similarly, the phylogenies presented in this paper provide quantitative support for the use of several taxa (denoted with asterisks in Figs. 1–3) as calibrators for particular crown clades in the Syngnathoidei.

In contrast, the use of the other oldest extinct syngnathoid †*Eekaulostomus cuevasae* from the Danian of Mexico, as a calibration for the Aulostomidae + Fistulariidae total clade (Cantalice and Alvarado-Ortega 2016; Santaquiteria et al. 2021; Stiller et al. 2022) is not justified based on the results of phylogenetic analysis. †*Eekaulostomus cuevasae* is always recovered closer to Syngnathidae than to the trumpetfish total clade, except for when molecular constraints are forced and parsimony analysis is used. This latter analysis is not useful for including †*Eekaulostomus cuevasae* as a calibration point for trumpetfishes and cornetfishes, as using constraints forces morphological patterns to match molecular patterns of cladogenesis (see Parham et al. 2012).

**Fig. 4:**
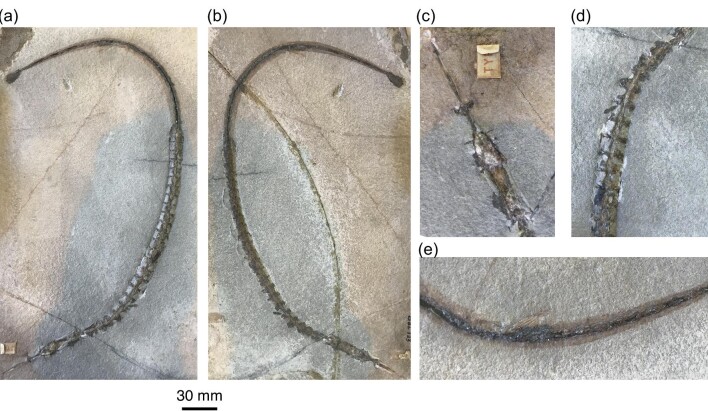
Photographs of the holotype specimen of †*Pseudosyngnathus opisthopterus*. Part (**A**) and counterpart (**B**) of specimen MNHN BOL 122, with details of (**C**) cranium, (**D**) partially armored thorax, and (**E**) anal fin. Scale bar refers to images in (a) and (b).

The phylogenetic hypothesis presented here serves as an example of how fossils can illuminate patterns of character change in a lineage that remain undetectable in samples that only include extant species (e.g., Luo 2007; Bever et al. 2011; Mongiardino Koch and Parry 2020; Mongiardino Koch et al. 2021; Brownstein et al. 2022). The reevaluation of fossil syngnathoid phylogeny presented here recasts †*Eekaulostomus cuevasae* as a transitional form from the partially armored or unarmored body plans of the trumpetfishes and shrimpfishes and the partially or completely developed dermal skeletons of ghost pipefishes (Solenostomidae) and pipefishes, seadragons, and seahorses (Syngnathidae). Although †*Eekaulostomus cuevasae* shares an elongated skull and thorax and opposed dorsal and anal fins with extant and fossil representatives of the Aulostomidae and Fistulariidae (Cantalice and Alvarado-Ortega 2016), its tube-like snout and proportionately long head are also reminiscent of the solenostomid genera *Solenostomus*, †*Calamostoma*, and †*Solenorhynchus*, as is its heavily armored skull and thorax. Whereas the heavily armored body of †*Eekaulostomus cuevasae* was previously thought to indicate a secondary loss of body armor in aulostomids and fistulariids, the new phylogenetic hypothesis for this taxon suggests it is illustrative of the variability in dermal skeleton ossification along the stem leading to syngnathids and solenostomids. This variance in exoskeleton development is also seen along the branch leading to the syngnathid crown in sympatric extinct species: †*Prosolenostomus* possesses a complete exoskeleton of heavily developed rectangular osteoderms similar to living seahorses, pipefishes, and seadragons, whereas †*Pseudosyngnathus opisthopterus* does not have a clearly developed exoskeleton along its caudal region (Fig. 4).

### The timescale of syngnathoid diversification

Because of their relatively rich fossil record for the Cretaceous-Eocene, Syngnathoidei is a key study lineage for timing the radiation of Acanthomorpha, which includes about one-third of living vertebrate diversity (Near et al. 2013; Alfaro et al. 2018; Rabosky et al. 2018; Dornburg and Near, 2021; Ghezelayagh et al. 2022). Due to the novel positions of several key fossils and the data type used (morphology), the phylogenetic trees generated in this study provide a new avenue toward resolving the timescale of syngnathoid and acanthomorph diversification. Although the Bayesian and parsimony analyses produce trees that are congruent with a view of Syngnathoidei as a primarily post-Cretaceous diversification (Alfaro et al. 2018; Santaquiteria et al. 2021; Cantalice et al. 2022; Stiller et al. 2022), the ages inferred for the major crown clades found in this study are often slightly younger than those inferred in node-dated phylogenomic trees (Santaquiteria et al. 2021; Stiller et al. 2022).

This moderate (Table 1) discrepancy in ages among the morphology-based tip-dated phylogenies presented here and previous phylogenomic trees may be due to (1) the revised phylogenetic positions of the two oldest syngnathoid fossils, †*Gasterorhamposus zuppichini* and †*Eekaulostomus cuevasae*, and (2) the fact that living species with currently somewhat unclear placements (e.g., *Doryichthys, Phyllopteryx;*Longo et al. 2017; Santaquiteria et al. 2021; Stiller et al. 2022) were allowed to drift across the tree, making divergence time estimates only partially comparable (Table 1). †*Eekaulostomus cuevasae* has been allied with Aulostomoidea based on only a handful of phylogenetically informative characters and is in some ways more similar to syngnathids and solenostomids. Thus, the placement of †*Eekaulostomus cuevasae* at the base of syngnathids may require less character state changes between this taxon and the crown, which reduces the ages of estimated divergences found using the uncorrelated lognormal clock. Any discrepancy in ages due to the differential placement of †*Eekaulostomus cuevasae* on the stem of Syngnathidae + Solenostomidae rather than Aulostomoidea as previously conducted should not be an issue when node dating is used for phylogenomic analyses, as positions of this taxon on the stem of either lineage as a node calibrator would mean that the same node (MRCA of crown Syngnathoidei) would be calibrated, and no morphological data would be included in the analysis.

### Toward a revised role of fossils in phylogenetics: syngnathoidei as a case study

In the era of phylogenomics (Smith et al. 2020), the role that fossils have to play in reconstructing evolutionary trees is being rethought. Whereas the early reliance on morphological datasets allowed fossils to be easily included in phylogenies of most extant clades (e.g., Estes et al. 1988), current approaches have emphasized the importance of fossils as tools for time-calibrating molecular phylogenies (e.g., Meredith et al. 2011; Near et al. 2012; Jones et al. 2013; Near et al. 2013; Jarvis et al. 2014; Prum et al. 2015; Gavryushkina et al. 2017; Alfaro et al. 2018; Burbrink et al. 2020; [Bibr bib18a][Bibr bib18a]) and for sampling extinct nodes that might stabilize character state transitions along morphological phylogenies (e.g., Gauthier et al. 2012; Reeder et al. 2015; Mongiardino Koch and Parry 2020; Mongiardino Koch et al. 2021). The rapid spread of fossil calibration usage has necessitated the construction of protocols for justifying fossil calibration placement and age (Parham et al. 2012). Although fossil calibrations are often rigorously justified in the literature, a problem exists when the phylogenetic positions of fossil calibrations themselves have not been tested using adequately taxon- and character-rich datasets. Syngnathoidei presents a key example of this phenomenon. Although phylogenomic studies have justified the placement of key early syngnathoids like †*Eekaulostomus cuevasae* based on published phylogenetic analyses (Cantalice and Alvarado-Ortega 2016; Murray 2022), increased sampling for both extant and fossil species presents a radically different hypothesis for the placement of this taxon that could affect the time calibration of the syngnathoid tree.

**Fig. 5:**
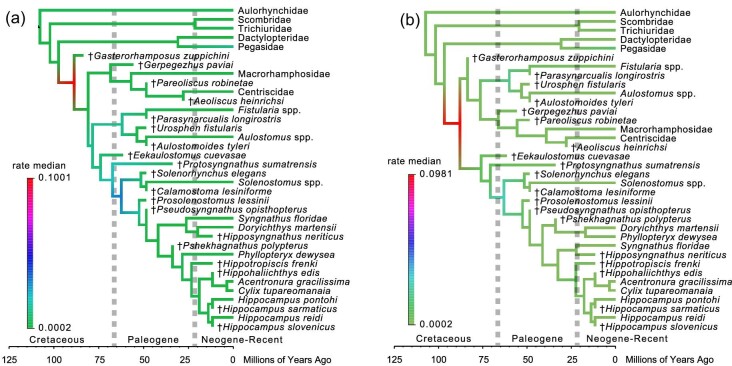
Tempo of syngnathoid phenotype evolution. Time-calibrated maximum clade credibility tree of syngnathoid interrelationships with median node times and constraints unenforced (**A**) or enforced (**B**) on living species interrelationships based on phylogenomic trees found using BEAST 2.6.6. values. Rates of morphological evolution (state changes per character per million years) are colored along branches. Note the increase in rate at the base of pan-Syngnathoidei and the small increase at the base of Solenostomidae + Syngnathidae. All photographs are public domain from Wikipedia Commons.

Morphological phylogenetic analyses are also important for providing a direct window into the tempo of phenotypic evolution. In particular, tests of the relationships of extinct species allow fossils to be put in a larger phylogenetic context and illuminate patterns of character change. Secondly, phylogenies constructed using morphological data can be used to directly reconstruct hypotheses of phenotypic evolutionary rates, particularly when fossils are included (e.g., Lloyd et al. 2012; Lee 2016; Wang and Lloyd 2016; Simões et al. 2020a, 2020b; Vernygora et al. 2020; Cui et al. 2022). Although the interrelationships of clades provide the fundamental framework to construct hypotheses about stepwise phenotypic evolution, the estimation of morphological evolutionary rates along a tree may provide a refined understanding of where major morphological rate shifts occur in an easily visualized manner. For example, although both the strict consensus (Fig. 1) and Bayesian maximum clade credibility trees (Figs. 2–4) of Syngnathoidei presented in this study suggest a stepwise acquisition of a complete exoskeleton in Syngnathidae from the tightly clustered scutes in †*Eekaulostomus cuevasae* (Cantalice and Alvarado-Ortega 2016) and the grid-like arrangement of stellate scutes in Solenostomidae (Bannikov and Carnevale 2017), estimations of morphological evolutionary rates (character state changes per million years) obtained from the unconstrained and constrained Bayesian analyses (Fig. 5) show that the major morphological rate shifts in Syngnathoidei occur at (1) the base of the syngnathoid total clade during the Late Cretaceous and (2) at the common ancestor of Solenostomidae and pan-Syngnathidae (Fig. 5); the second shift is much less pronounced (Fig. 5A and B) and is rendered even more subtle when topological constraints are enforced based on the phylogenomic hypothesis of syngnathoid interrelationships (Fig. 5B). These results agree with a recent study of acanthomorph morphological evolution, which found evidence for a protracted disparification following the Cretaceous-Paleogene mass extinction (Ghezelayagh et al. 2022).

## Conclusions

Syngnathoidei is a morphologically disparate, speciose clade of acanthomorph fishes that represents a key model clade for studying the tempo and mode of phenotypic innovation. By reconstructing the first comprehensive phylogenetic hypothesis of the relationships of fossil syngnathoids using morphological data, I suggest novel placements for key early fossil syngnathoids that may affect future attempts to time-calibrate syngnathoid phylogeny. Further, I use the new reconstructions of the phylogeny of living and extinct syngnathoids to demonstrate the importance of using multiple analytical protocols (Bayesian versus parsimony, constrained versus unconstrained) to test the placement of key fossil calibrators and reconstruct the tempo of morphological evolution.

## Supplementary Material

obad011_Supplemental_Files

## Data Availability

All data is available in the supplementary material uploaded with this article.
