# Supplementary material for: Syngnathoid Evolutionary History and the Conundrum of Fossil Misplacement
Source: Integr Org Biol. 2023 May 4;5(1):obad011. doi: 10.1093/iob/obad011 (PMC10210065; doi:10.1093/iob/obad011)
Supplement: obad011_Supplemental_Files [file obad011_supplemental_files.zip › Supplemental Da.docx]

**Supplement To: Syngnathoid evolutionary history and the conundrum of fossil misplacement**

**Appendix 1.** Note on Monte Bolca specimens examined.

I personally examined all holotypes of Monte Bolca syngnathoid specimens housed at the French National Museum of Natural History (Musee National de Histoire Naturelle Paris; MNHN). One specimen, MNHN BOL 560, was previously referred to *Syngnathus* sp. Examination of this specimen under the microscope reveals it has a complete exoskeleton, a heavily reduced caudal fin, and no pelvic fins, all of which are syngnathid apomorphies. Examination of the skull shows it possesses a relatively short rostrum and what appears to be a tenticular crest, both of which are key features of the species †*Prosolenostomus lessini* (Bannikov and Carnevale, 2017). As such, this specimen can be assigned to that taxon, and is the third such skeleton of this species identified.

**Appendix 2.** List of characters and states modified from Murray (2022).

1. Nasal bone: 0) present; 1) absent.
2. Lateral ethmoid: 0) confined to forming the anterior edge of the orbit; 1) forming part of the medial separation between orbits.
3. Vomer connection with lateral ethmoids: 0) head of vomer meets lateral ethmoids; 1) head of vomer does not meet lateral ethmoids.
4. Vomerine teeth: 0) absent; 1) present.
5. Vomer length: 0) shaft extends posteriorly past lateral ethmoid; 1) shaft ends anterior to the posterior tips of lateral ethmoids.
6. Infraorbital bones: 0) form a complete ring ventral to the orbit from the lateral ethmoid to the dermosphenotic; 1) do not form a complete ventral ring (there is a gap between the last infraorbital bone and the dermosphenotic).
7. Frontal canals: 0) supraorbital canal present on frontal; 1) no supraorbital canal on frontal.
8. Frontal and parasphenoid: 0) frontal does not meet lateral process of parasphenoid; 1) frontal meets lateral process of parasphenoid.
9. Parietals: 0) present; 1) absent.
10. Intercalar: 0) present; 1) absent.
11. Prootic and exoccipital: 0) contact one another; 1) do not contact, separated by pterotic.
12. Prootic and basioccipital: 0) contact one another; 1) do not contact (separated by the pterotic or a large gap in ossification).
13. Pterotic and basioccipital: 0) contact one another; 1) do not contact, they are separated by the exoccipital.
14. Basisphenoid: 0) present; 1) absent.
15. Pterosphenoid: 0) present; 1) absent.
16. Parasphenoid width: 0) not expanded between lateral ethmoids; 1) expanded between lateral ethmoids.
17. Parasphenoid shaft: 0) forked posteriorly, posterior end ‘divided’; 1) pointed posteriorly, posterior end ‘not divided’.
18. Supraoccipital crest: 0) present; 1) absent.
19. Jaw teeth 0) present; 1) absent.
20. Premaxilla ascending process: 0) absent or greatly reduced, 1) present and prominent. Codings checked, as the states were reversed in the matrix of Murray (2022).
21. Premaxilla articular process: 0) present; 1) absent.
22. Maxilla shaft: 0) not expanded 1) greatly expanded ventrally. Codings checked, as the states were reversed in the matrix of Murray (2022).
23. Palatine teeth: 0) present; 1) absent.
24. Palatine head: 0) cylindrical; 1) laterally compressed.
25. Endopterygoid and ectopterygoid: 0) separate elements; 1) represented by a single element (it is assumed the two are fused together).
26. Metapterygoid represented by a separate element: 0) present; 1) absent (fused to hyomandibula or symplectic). Character was rewritten.
27. Metapterygoid position in relation to orbit: 0) posterior to orbit; 1) anterior to the orbit. Character was rewritten.
28. Quadrate shape: 0) more or less fan-shaped; 1) elongated, bearing a deep dorsal flange.
29. Quadrate shaft length: 0) about the same length as quadrate body; 1) much longer than quadrate body.
30. Quadrate anterodorsal expansion: 0) absent, shaft of quadrate of similar depth along length; 1) lamina of bone reaches dorsally at anterior end of quadrate.
31. Articulation of quadrate and lower jaw: 0) positioned below the orbit; 1) positioned in front of orbit.
32. Sympletic flanges: 0) absent, symplectic is of roughly similar depth along its length; 1) present, symplectic expanded by lamina of bone.
33. Symplectic anterior end: 0) not bifurcated; 1) bifurcated.
34. Preopercle articular socket for interhyal: 0) absent; 1) present.
35. Hyomandibula dorsal head: 0) with two separate condyles for articulation with skull; 1) single head (condyles fused together or indistinct).
36. Preopercle dorsal and ventral limbs: 0) distinct limbs present and of roughly equal size; 1) dorsal and ventral limbs are not clearly demarcated from one another.
37. Preopercle sensory canal: 0) enclosed in bone; 1) runs in open trough.
38. Subopercle ascending anterior limb: 0) present, subopercle rises dorsally at the anterior corner between opercle and preopercle; 1) absent, subopercle does not separate ventral portions of the opercle and preopercle.
39. Subopercle and interopercle: 0) close together; 1) widely separated from each other.
40. Interopercle length: 0) short, not extending full length of preopercle; 1) elongated, extending full length of preopercle.
41. Opercle dorsal projection: 0) present; bone extends dorsal to level of articulation with hyomandibula; 1) absent; opercle is flattened or truncated dorsally, not extending dorsally beyond level of articulation with hyomandibula.
42. Basihyal: 0) small; 1) elongated.
43. Urohyal ventrolateral flange: 0) absent; 1) present.
44. Urohyal blade: 0) entire; 1) incised posteriorly.
45. Hypohyals: 0) dorsal and ventral hypohyals of similar size and articulate on anterior end of anterior ceratohyal; 1) ventral hypohyal much larger than dorsal hypohyal, with dorsal hypohyal articulating more posteriorly on anterior ceratohyal than ventral hypohyal.
46. Anterior ceratohyal shape: 0) bone becomes abruptly narrower anteriorly; 1) bone is of similar depth along its length or only gradually becomes narrower anteriorly.
47. Anterior and posterior ceratohyal connection: 0) struts of bone present on one element overlap the other; 1) no struts of bone overlap the elements, the two are joined by connective tissue only.
48. Interhyal shape: 0) cylindrical or triangular, not sutured to posterior ceratohyal; 1) flat and round, sutured to posterior ceratohyal.
49. Branchiostegal ray number: 0) more than four; 1) four or fewer.
50. Branchiostegal rays articulating on posterior ceratohyal: 0) one or more branchiostegals articulate on posterior ceratohyal; 1) none of the branchiostegal rays articulate on the posterior ceratohyal.
51. Gill membrane: 0) free from isthmus; 1) united to isthmus.
52. Ossified basibranchials: 0) three; 1) fewer than three.
53. Hypobranchial number: 0) three; 1) fewer than three.
54. Epibranchial 3 and 4 uncinate processes: 0) present; 1) absent.
55. Epibranchial 4: 0) present; 1) absent.
56. Pharyngobranchial 1: 0) present; 1) absent.
57. Pharyngobranchial 2 tooth plate: 0) present; 1) absent.
58. Pharnygobranchial 4 tooth plate: 0) present; 1) absent.
59. Interarcual cartilage: 0) absent; 1) present.
60. Gill filament shape: 0) elongate; 1) lophobranch, arranged in rounded tufts.
61. Branchial arch tooth plates: 0) present; 1) absent.
62. Posttemporal dorsal process attachment to epioccipital: 0) tightly attached by short ligament to epioccipital; 1) co-ossified with cranium.
63. Posttemporal sensory canal: 0) absent; 1) present.
64. Supracleithrum: 0) present, fairly large; 1) reduced or absent.
65. Cleithrum dorsal plate: 0) with pointed anterodorsal process; 1) lacking an anterodorsal process.
66. Cleithrum ventral limb: 0) undivided; 1) divided into two struts.
67. Cleithrum posteromedial extension towards coracoids: 0) absent; 1) present.
68. Postcleithra number: 0) one; 1) none.
69. Scapular foramen: 0) complete, contained fully in scapula; 1) incomplete, opens to edge of scapula.
70. Scapula and first actinost (pectoral radial): 0) separate from one another; 1) closely abutting one another; 2) assumed to be fused together (only three separate actinosts present).
71. Coracoid posterior extension: 0) absent, coracoid does not reach further posteriorly than actinosts; 1) present, coracoid reaches past level of actinosts.
72. Ectocoracoid: 0) absent; 1) present.
73. Pectoral actinost shape: 0) unconstricted, roughly rectangular; 1) strongly constricted centrally to form an hourglass-shape.
74. Pectoral actinost length: 0) actinosts short, not much longer than wide; 1) actinosts elongate, longer than wide.
75. Pectoral actinost 4, anteroventral spike: 0) absent; 1) present.
76. Pelvic fins: 0) present; 1) absent.).
77. Pelvic spines: 0) present; 1) absent.
78. Pelvic girdle suturing: 0) left and right plates are separate from one another or meet in a straight edge which lacks a suture; 1) left and right plates are joined by medial interdigitating suture.
79. Pelvic girdle median posterior process: 0) not well developed, not extending much past level of ray articulation; 1) well developed, extending as a clear process further posteriorly than level of ray articulation.
80. Second dorsal fin spines: 0) present; 1) absent.
81. Anal fin spines: 0) present; 1) absent.
82. Anterior vertebrae fusion: 0) first few centra separate from one another (although they may have a few interdigitating sutures; 1) first few centra fused together.
83. Anterior centrum size: 0) similar to other abdominal centra in size; 1) first one to few anterior centra distinctly longer than more posterior ones.
84. Transverse processes of first two vertebrae: 0) present; 1) absent.
85. Anterior vertebrae and scute connection: 0) vertebrae lack lateral process to scutes; 1) vertebrae bear lateral processes that connect to scutes.
86. Neural arch of precaudal vertebrae: 0) not pierced by foramina; 1) pierced by foramina.
87. Neurohypophyses: 0) present; 1) absent.
88. Epipleurals: 0) present; 1) absent.
89. Bony scutes: 0) absent; 1) present.
90. Parhypural: 0) autogenous; 1) fused to preural centrum.
91. Hypural fusion: 0) autogenous; 1) all fused to the centrum.
92. Number of hypural plates: 0) three or more; 1) two; 2) one.
93. Neural spine of second preural centrum length: 0) shorter than other neural spines; 1) similar size as other neural spines.
94. NEW Tail anatomy: 0) Not prehensile; 1) prehensile. A prehensile tail is an apomorphy of Hippocampine, *Nerophis* + *Entelurrus* and Solegnathini (Neutens et al., 2014).


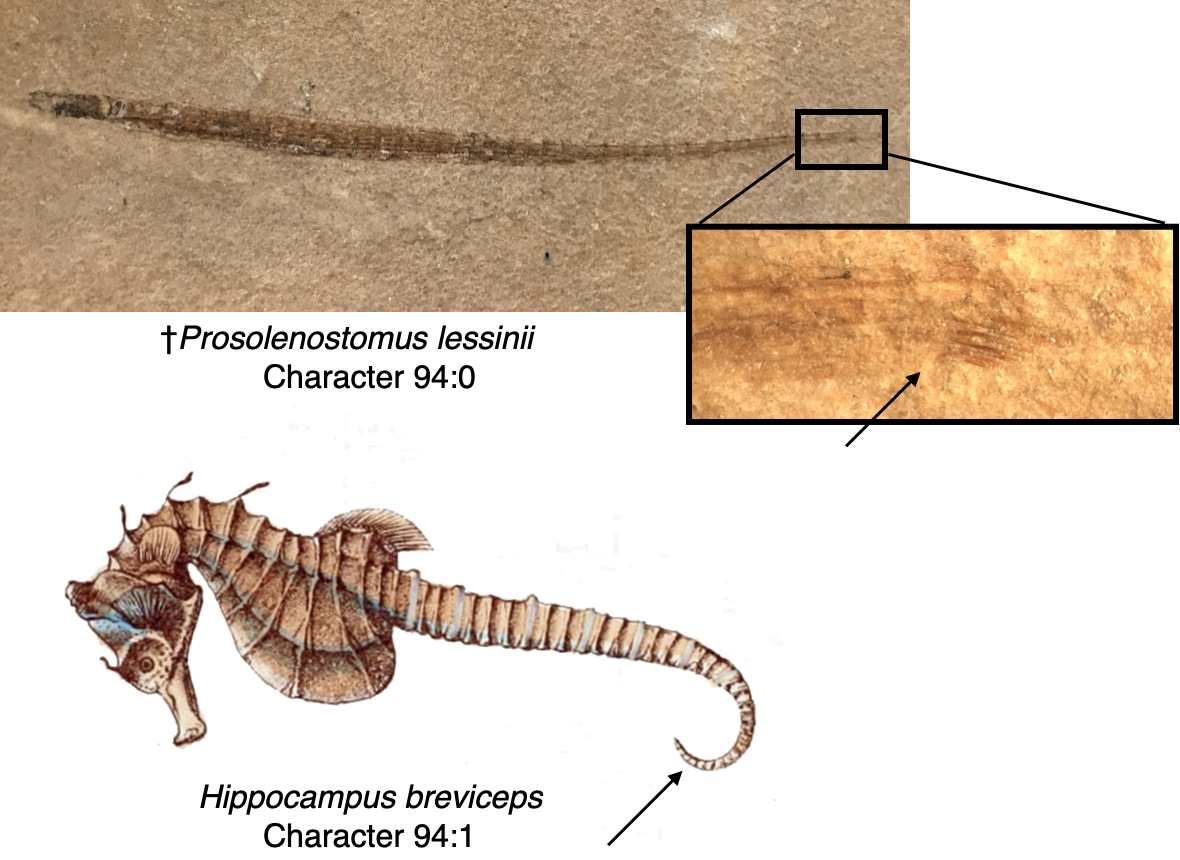


1. NEW Position of pelvic fin insertion: 0) directly ventral to spinous dorsal fin; 1) anterior to spinous dorsal fin. State 0 is an apomorphy of †*Calamostoma* + †*Solenorhynchus* (see Bannikov and Carnevale, 2017) and represents a character state reversal from the *Solenostoma* state (state 1).


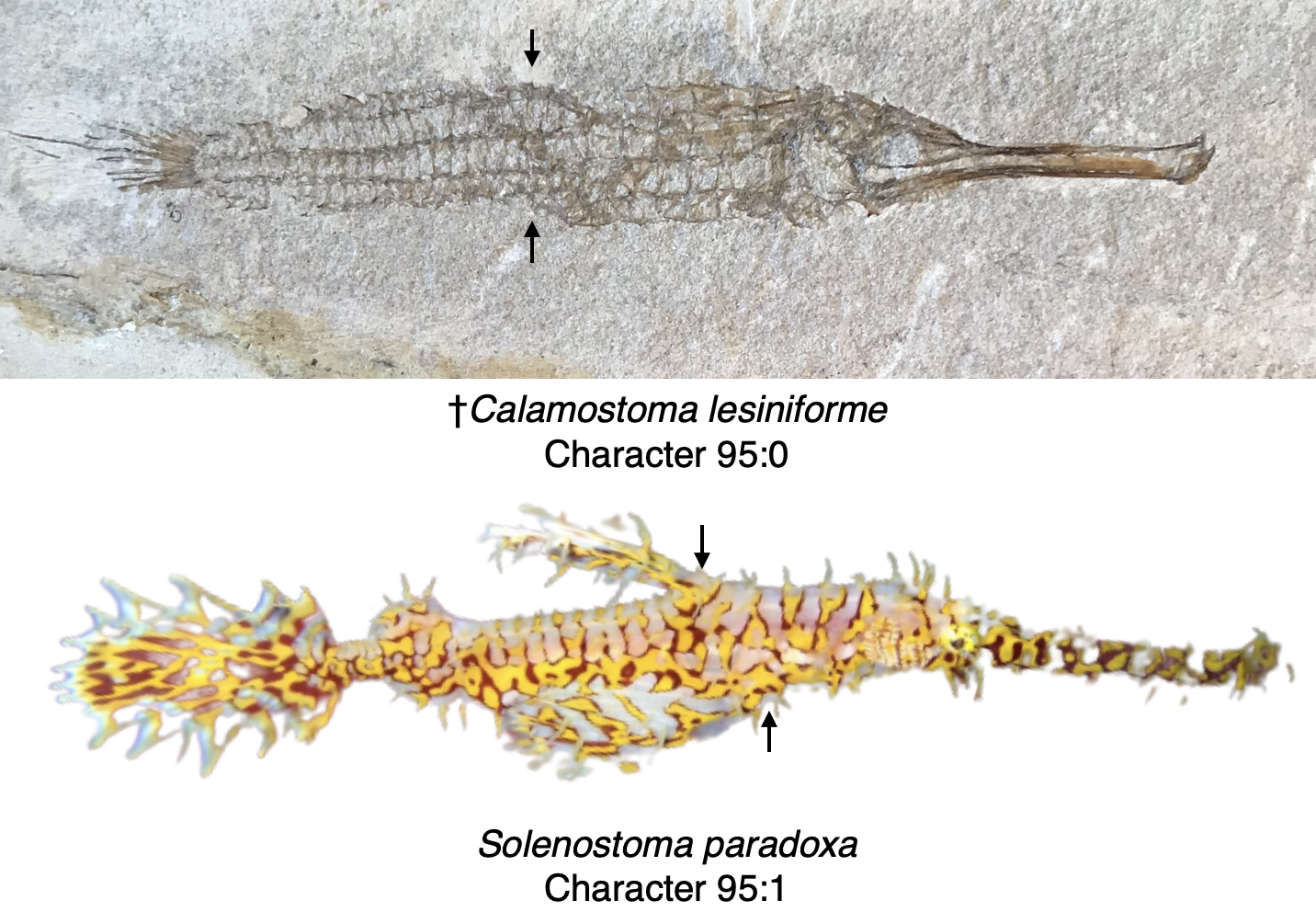


1. NEW Posture: 0) Horizontal, head and body main axis approximately straight; 1) head main axis slightly offset from body to form wide angle (approximately 25°); 2) upright, head and body axes at acute angle (slightly less than or equal to 90°). State 1 is found in solenostomids, stigmatophorins, and pygmy pipehorses (Haliichthyini and *Amphelikturus dendriticus*). State 2 refers to the upright condition in true seahorses (Hippocampini).


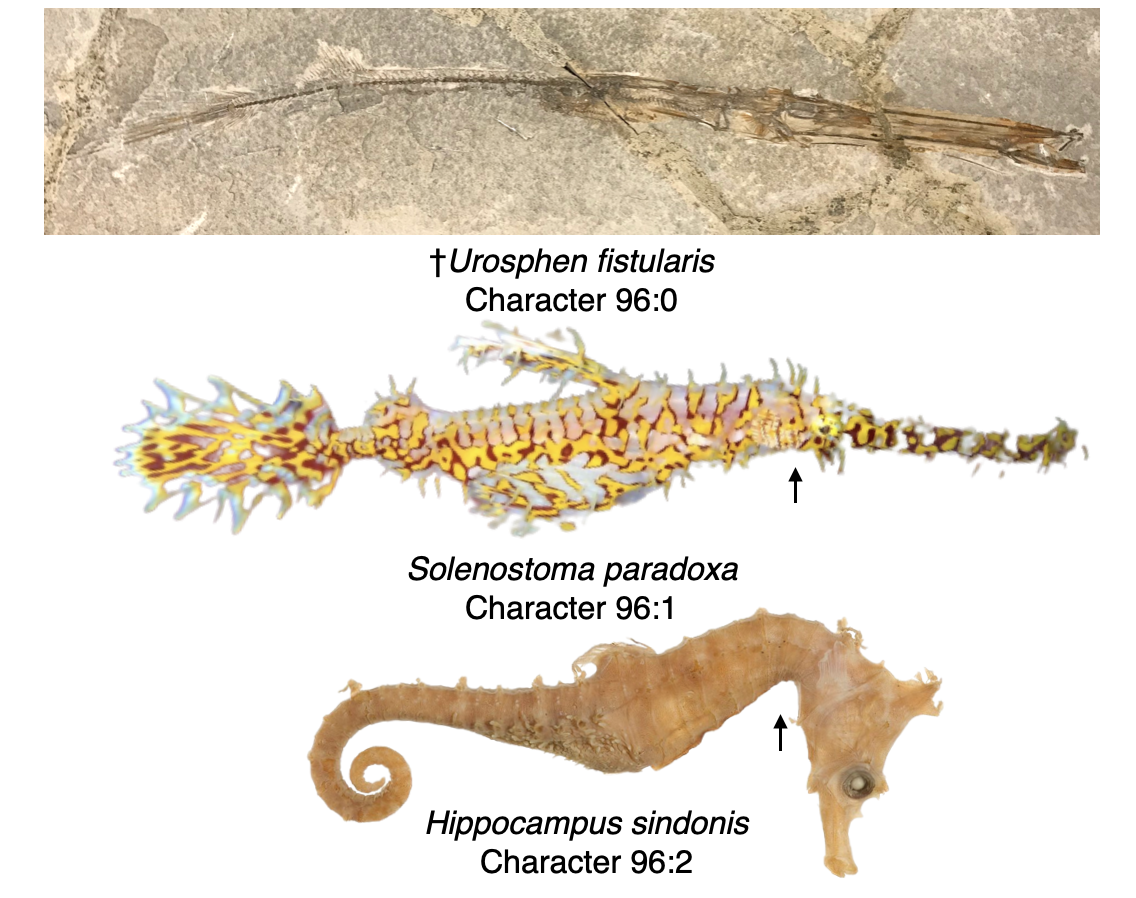


1. NEW Gill openings: 0) paired; 1) single on midline. 0 is the state in non-pygmy seahorses and other fishes. 1 is the state in pygmy seahorses. See Short et al. (2018), *Zookeys* 779:27-49.
2. NEW Dorsal fin base: 0) Flattened, in line with rest of body; 1) raised. State 1 is an apomorphy of *Hippocampus* and pygmy pipehorses in the clade Haliichthyini. *Amphelikturus dendriticus,* which is not recovered in Haliichthyini (Stiller et al., 2022), may have this condition.


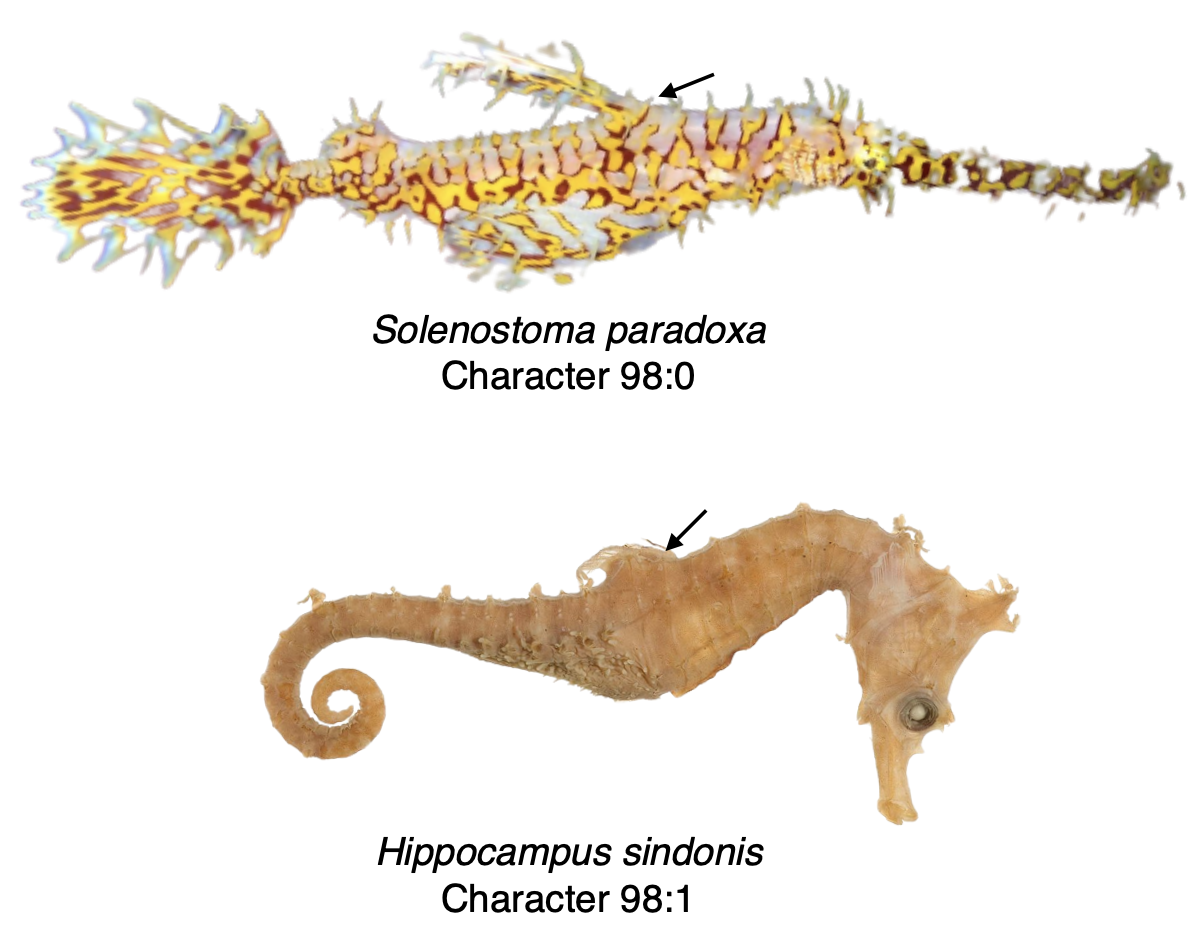


1. NEW Caudal fin: 0) Present; 1) absent. State 1 is an apomorphy of *Hippocampus* and pygmy pipehorses in the clade Haliichthyini, as well as *Nerophis*+*Entelurrus* and Solegnathini (Neutens et al., 2014).


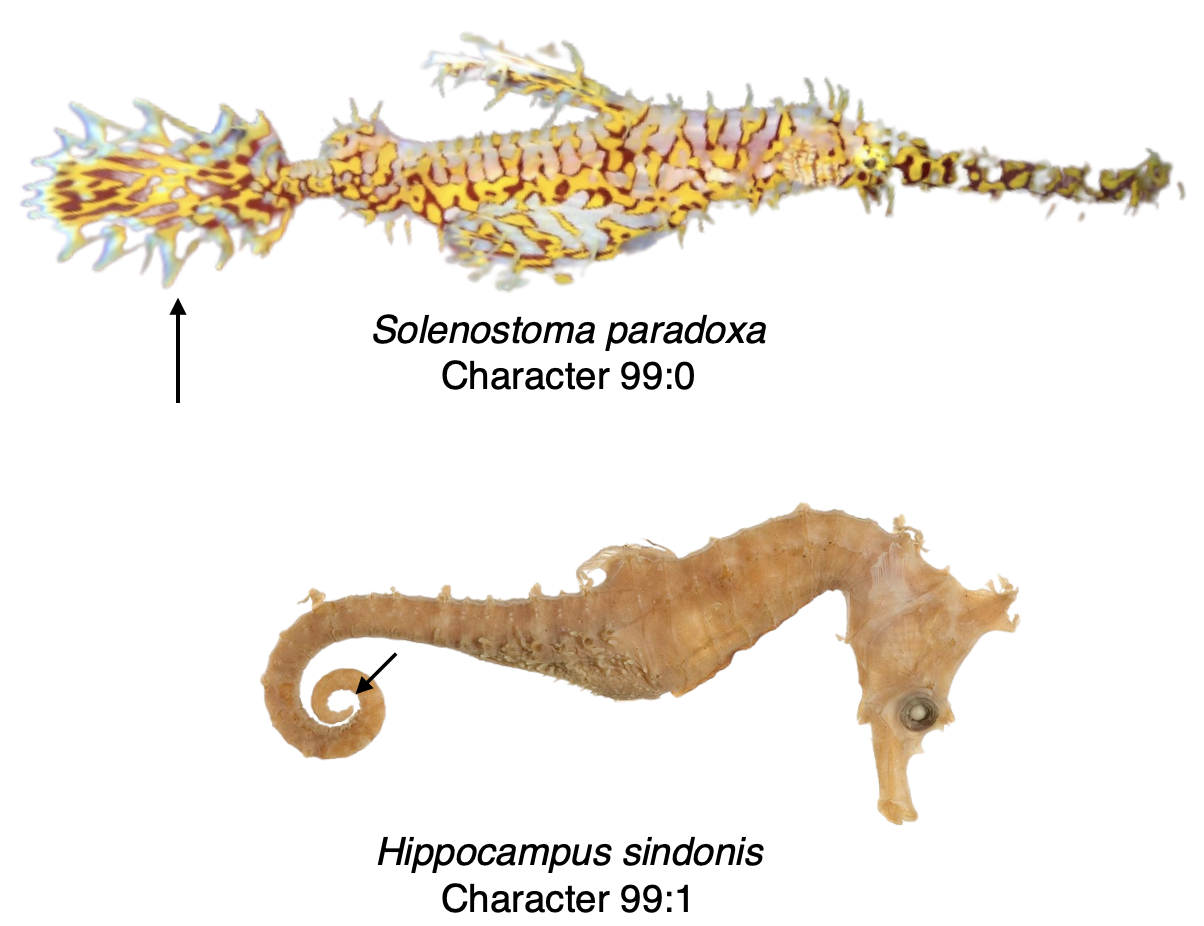


1. NEW Bony exoskeleton: 0) Absent; 1) partial, large gaps between stellate elements; 2) complete, extensive fusion and little to no gaps between scute elements.


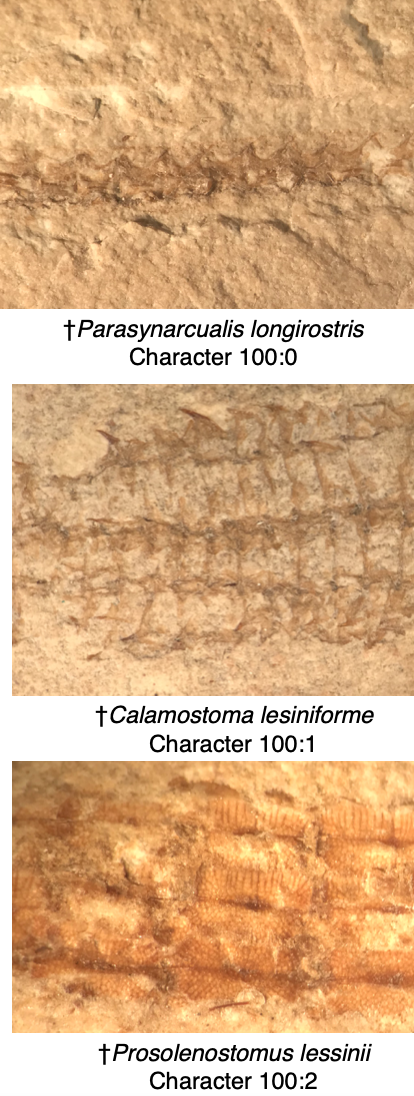


1. NEW Brooding pouch location: 0) Absent or contained in pelvic fins; 1) located on trunk; 2) located on tail. 1 is the trait in Nerophinae, 2 is the trait in Sygnathinae.

Note: all images of specimens above are by the author or public domain from the Smithsonian or Wikimedia commons.

**Appendix 3.** Unconstrained parsimony analysis results.

Figure S1. Tree Showing Nodes for Apomorphy List.


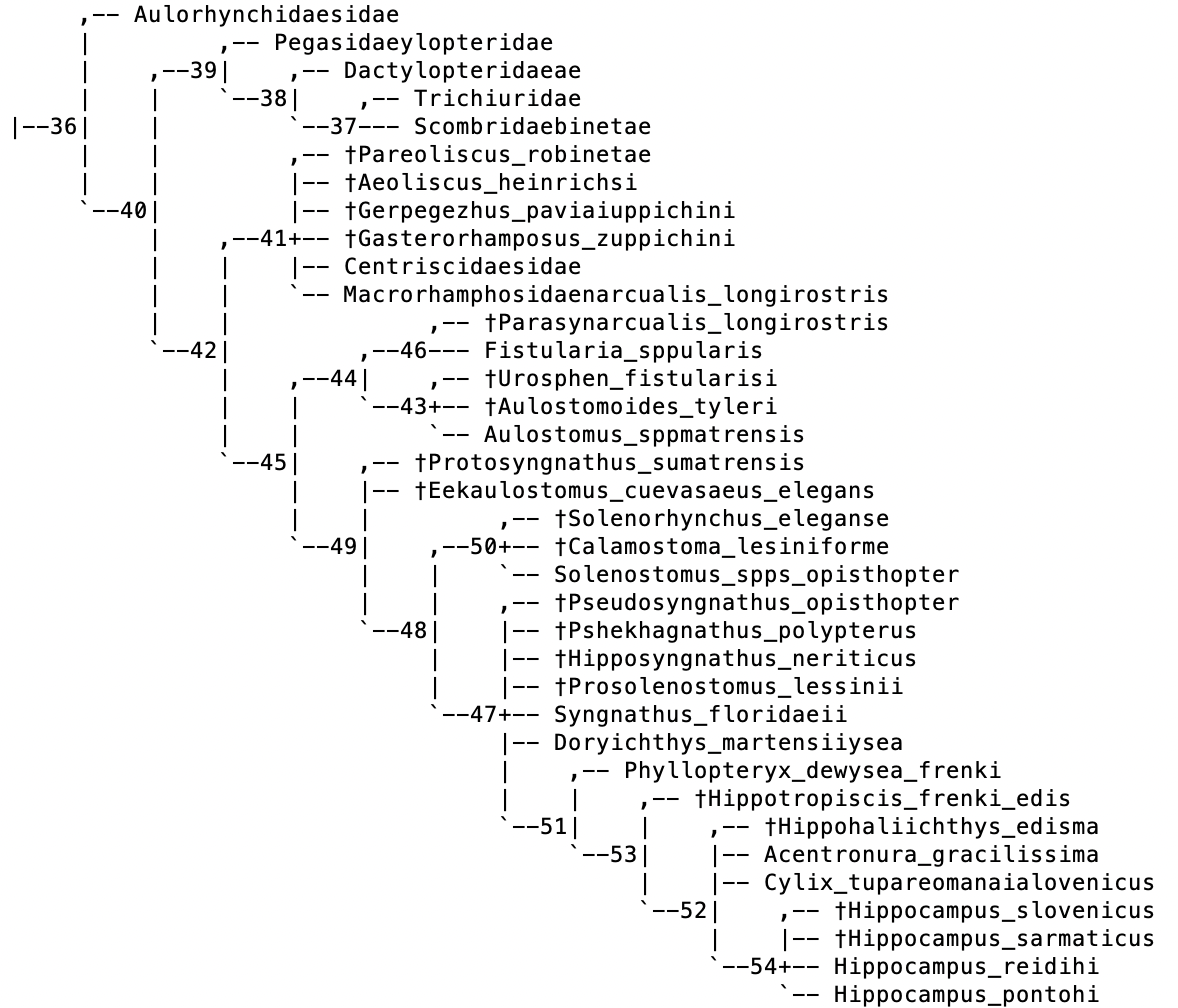


Most Parsimonious Trees Generated: 315.

Tree Length: 216.

Consistency Index: 0.486.

Retention Index: 0.777.

Apomorphy List (TNT Numbering, char 1 = char 0 below, etc.):

Aulorhynchidae :

No autapomorphies

Scombridae :

Char. 22: 1 → 0

Trichiuridae :

Char. 2: 0 → 1

Char. 31: 0 → 1

Char. 35: 0 → 1

Char. 75: 0 → 1

Pegasidae :

Char. 1: 0 → 1

Char. 18: 0 → 1

Char. 23: 0 → 1

Char. 25: 0 → 1

Char. 27: 0 → 1

Char. 35: 0 → 1

Char. 51: 0 → 1

Char. 52: 0 → 1

Char. 53: 0 → 1

Char. 59: 0 → 1

Char. 69: 0 → 2

Dactylopteridae :

Char. 4: 0 → 1

Char. 20: 1 → 0

Char. 63: 0 → 1

Char. 67: 1 → 0

Char. 73: 0 → 1

Char. 83: 0 → 1

Macrorhamphosidae :

Char. 4: 0 → 1

Char. 31: 0 → 1

Char. 61: 1 → 0

Char. 63: 1 → 0

Char. 70: 1 → 0

Char. 78: 0 → 1

Char. 80: 1 → 0

Centriscidae :

Char. 23: 0 → 1

Char. 25: 0 → 1

Char. 55: 0 → 1

Char. 81: 0 → 1

Char. 89: 1 → 0

Aulostomus_spp :

Char. 69: 0 → 1

Fistularia_spp :

No autapomorphies

Doryichthys_martensii :

No autapomorphies

Solenostomus_spp :

Char. 94: 1 → 0

Phyllopteryx_dewysea :

Char. 79: 1 → 0

Char. 80: 1 → 0

Syngnathus_floridae :

No autapomorphies

Cylix_tupareomanaia :

No autapomorphies

Acentronura_gracilissima :

No autapomorphies

Hippocampus_pontohi :

Char. 96: 0 → 1

Char. 100: 2 → 1

Hippocampus_reidi :

No autapomorphies

†Aulostomoides_tyleri :

No autapomorphies

†Urosphen_fistularis :

Char. 34: 0 → 1

†Parasynarcualis_longirostris :

No autapomorphies

†Eekaulostomus_cuevasae :

Char. 80: 1 → 0

†Protosyngnathus_sumatrensis :

Char. 4: 0 → 1

Char. 36: 1 → 0

Char. 93: 0 → 1

†Hippotropiscis_frenki :

No autapomorphies

†Prosolenostomus_lessinii :

Char. 7: 0 → 1

†Hipposyngnathus_neriticus :

Char. 75: 1 → 0

†Hippohaliichthys_edis :

No autapomorphies

†Pshekhagnathus_polypterus :

No autapomorphies

†Hippocampus_sarmaticus :

No autapomorphies

†Hippocampus_slovenicus :

No autapomorphies

†Calamostoma_lesiniforme :

No autapomorphies

†Solenorhynchus_elegans :

No autapomorphies

†Gasterorhamposus_zuppichini :

Char. 11: 1 → 0

†Pseudosyngnathus_opisthopter :

No autapomorphies

†Gerpegezhus_paviai :

Char. 4: 0 → 1

Char. 8: 1 → 0

Char. 64: 1 → 0

Char. 75: 0 → 1

†Aeoliscus_heinrichsi :

No autapomorphies

†Pareoliscus_robinetae :

No autapomorphies

Node 37 :

Char. 6: 0 → 1

Char. 7: 1 → 0

Char. 15: 1 → 0

Char. 19: 0 → 1

Char. 21: 1 → 0

Char. 40: 1 → 0

Char. 45: 0 → 1

Char. 80: 1 → 0

Node 38 :

Char. 36: 1 → 0

Char. 68: 1 → 0

Char. 85: 1 → 0

Char. 89: 1 → 0

Char. 90: 1 → 0

Char. 91: 2 → 1

Node 39 :

Char. 2: 1 → 0

Char. 13: 1 → 0

Char. 30: 1 → 0

Char. 39: 1 → 0

Char. 56: 0 → 1

Char. 57: 0 → 1

Char. 70: 1 → 0

Char. 86: 1 → 0

Char. 92: 1 → 0

Node 40 :

No synapomorphies

Node 41 :

Char. 18: 0 → 1

Char. 34: 0 → 1

Char. 47: 0 → 1

Char. 49: 0 → 1

Char. 64: 0 → 1

Char. 68: 1 → 0

Char. 74: 0 → 1

Node 42 :

Char. 5: 0 → 1

Char. 8: 0 → 1

Char. 10: 0 → 1

Char. 11: 0 → 1

Char. 12: 1 → 0

Char. 21: 1 → 0

Char. 26: 0 → 1

Char. 28: 0 → 1

Char. 33: 0 → 1

Char. 42: 1 → 0

Char. 44: 0 → 1

Char. 67: 1 → 0

Char. 76: 0 → 1

Char. 77: 1 → 0

Node 43 :

Char. 60: 0 → 1

Char. 64: 0 → 1

Node 44 :

Char. 58: 0 → 1

Char. 65: 0 → 1

Char. 73: 0 → 1

Char. 81: 0 → 1

Char. 88: 1 → 0

Char. 91: 2 → 1

Node 45 :

Char. 43: 1 → 0

Char. 51: 0 → 1

Char. 53: 0 → 1

Char. 54: 0 → 1

Node 46 :

Char. 13: 1 → 0

Node 47 :

Char. 7: 1 → 0

Char. 34: 0 → 1

Char. 36: 1 → 0

Char. 56: 0 → 1

Char. 57: 0 → 1

Char. 64: 0 → 1

Char. 75: 0 → 1

Char. 82: 1 → 0

Char. 100: 0 → 12

Node 48 :

Char. 6: 0 → 1

Char. 18: 0 → 1

Char. 37: 0 → 1

Char. 39: 1 → 0

Char. 67: 0 → 1

Node 49 :

Char. 0: 0 → 1

Char. 27: 0 → 1

Char. 29: 0 → 1

Char. 99: 0 → 12

Node 50 :

Char. 65: 0 → 1

Char. 66: 1 → 0

Char. 76: 1 → 0

Node 51 :

Char. 95: 0 → 1

Char. 98: 0 → 1

Node 52 :

Char. 93: 0 → 1

Node 53 :

Char. 97: 0 → 1

Node 54 :

Char. 95: 1 → 2

**Appendix 4.** Constrained parsimony analysis results.


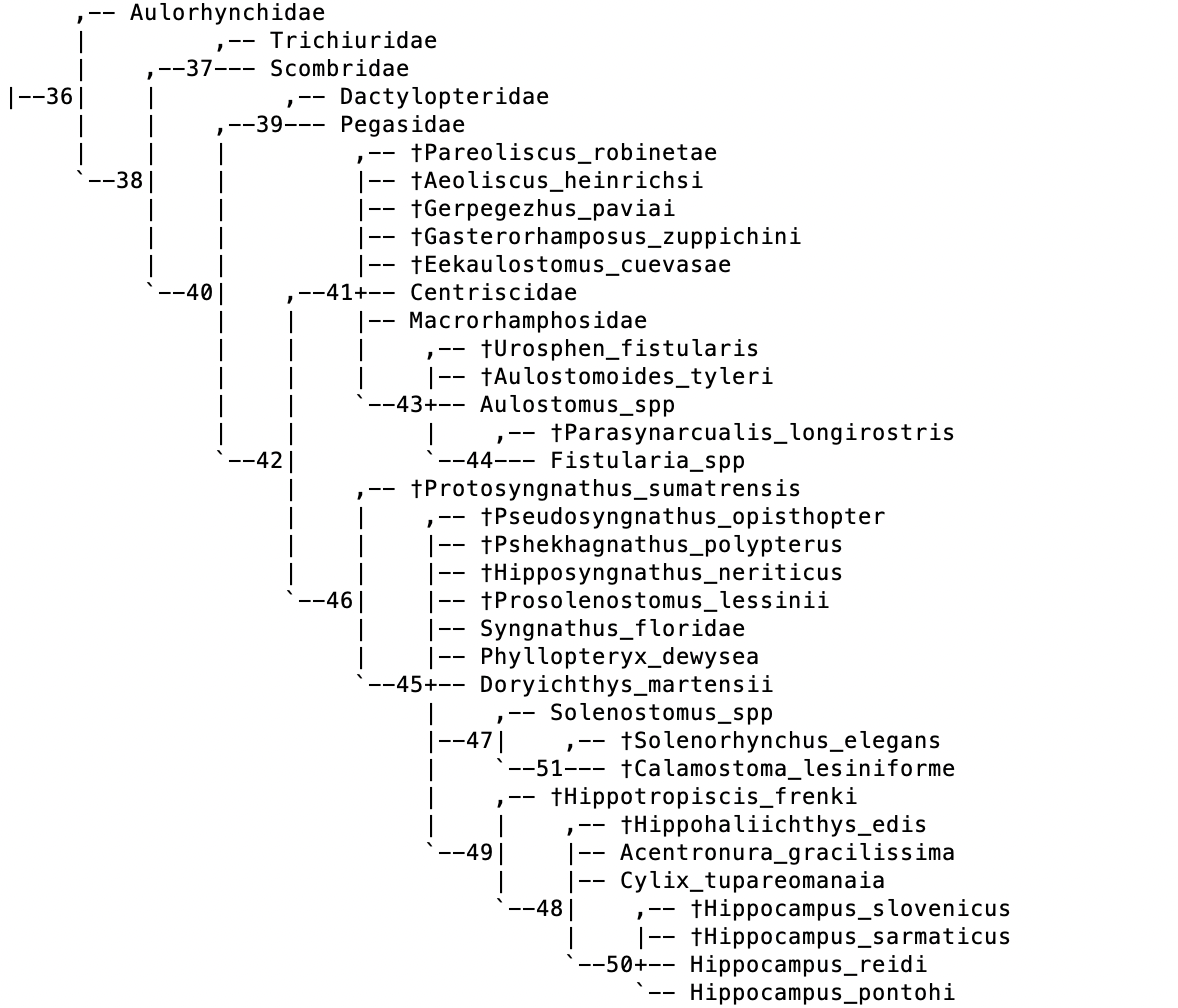
Figure S2. Tree Showing Nodes for Apomorphy List.

Most Parsimonious Trees Generated: >100,000.

Tree Length: 216.

Consistency Index: 0.459.

Retention Index: 0.751.

Apomorphy List (TNT Numbering, char 1 = char 0 below, etc.):

Aulorhynchidae :

No autapomorphies

Scombridae :

Char. 2: 1 → 0

Char. 22: 1 → 0

Trichiuridae :

Char. 17: 0 → 1

Char. 31: 0 → 1

Char. 35: 0 → 1

Char. 75: 0 → 1

Pegasidae :

Char. 1: 0 → 1

Char. 16: 1 → 0

Char. 23: 0 → 1

Char. 24: 0 → 1

Char. 25: 0 → 1

Char. 27: 0 → 1

Char. 35: 0 → 1

Char. 51: 0 → 1

Char. 52: 0 → 1

Char. 53: 0 → 1

Char. 69: 0 → 2

Char. 94: 0 → 1

Dactylopteridae :

Char. 4: 0 → 1

Char. 17: 0 → 1

Char. 20: 1 → 0

Char. 67: 1 → 0

Char. 73: 0 → 1

Char. 83: 0 → 1

Char. 85: 1 → 0

Char. 89: 1 → 0

Char. 90: 1 → 0

Char. 91: 2 → 1

Macrorhamphosidae :

Char. 4: 0 → 1

Char. 31: 0 → 1

Char. 50: 1 → 0

Char. 61: 1 → 0

Char. 63: 1 → 0

Char. 69: 0 → 1

Char. 70: 1 → 0

Char. 78: 0 → 1

Char. 80: 1 → 0

Centriscidae :

Char. 23: 0 → 1

Char. 25: 0 → 1

Char. 55: 0 → 1

Char. 66: 1 → 0

Char. 69: 0 → 2

Char. 81: 0 → 1

Char. 85: 1 → 0

Char. 89: 1 → 0

Aulostomus_spp :

Char. 45: 0 → 1

Char. 48: 0 → 1

Char. 62: 0 → 1

Char. 69: 0 → 1

Fistularia_spp :

No autapomorphies

Doryichthys_martensii :

Char. 100: 2 → 1

Solenostomus_spp :

No autapomorphies

Phyllopteryx_dewysea :

Char. 79: 1 → 0

Char. 80: 1 → 0

Char. 95: 0 → 1

Char. 98: 0 → 1

Syngnathus_floridae :

No autapomorphies

Cylix_tupareomanaia :

No autapomorphies

Acentronura_gracilissima :

No autapomorphies

Hippocampus_pontohi :

Char. 96: 0 → 1

Char. 100: 2 → 1

Hippocampus_reidi :

No autapomorphies

†Aulostomoides_tyleri :

No autapomorphies

†Urosphen_fistularis :

Char. 34: 0 → 1

†Parasynarcualis_longirostris :

No autapomorphies

†Eekaulostomus_cuevasae :

Char. 27: 0 → 1

Char. 29: 0 → 1

Char. 64: 1 → 0

Char. 71: 0 → 1

Char. 80: 1 → 0

Char. 99: 0 → 1

†Protosyngnathus_sumatrensis :

Char. 4: 0 → 1

Char. 93: 0 → 1

†Hippotropiscis_frenki :

No autapomorphies

†Prosolenostomus_lessinii :

Char. 7: 0 → 1

†Hipposyngnathus_neriticus :

Char. 75: 1 → 0

Char. 100: 2 → 1

†Hippohaliichthys_edis :

No autapomorphies

†Pshekhagnathus_polypterus :

No autapomorphies

†Hippocampus_sarmaticus :

No autapomorphies

†Hippocampus_slovenicus :

No autapomorphies

†Calamostoma_lesiniforme :

No autapomorphies

†Solenorhynchus_elegans :

No autapomorphies

†Gasterorhamposus_zuppichini :

Char. 11: 1 → 0

†Pseudosyngnathus_opisthopter :

No autapomorphies

†Gerpegezhus_paviai :

Char. 4: 0 → 1

Char. 8: 1 → 0

Char. 64: 1 → 0

Char. 75: 0 → 1

†Aeoliscus_heinrichsi :

Char. 66: 1 → 0

Char. 85: 1 → 0

†Pareoliscus_robinetae :

No autapomorphies

Node 37 :

Char. 6: 0 → 1

Char. 19: 0 → 1

Char. 40: 1 → 0

Char. 85: 1 → 0

Char. 88: 1 → 0

Node 38 :

No synapomorphies

Node 39 :

Char. 2: 1 → 0

Char. 84: 1 → 0

Node 40 :

Char. 15: 0 → 1

Char. 45: 1 → 0

Char. 50: 0 → 1

Char. 79: 0 → 1

Char. 80: 0 → 1

Char. 82: 0 → 1

Node 41 :

Char. 16: 1 → 0

Char. 67: 1 → 0

Char. 70: 0 → 1

Char. 92: 0 → 1

Char. 94: 0 → 1

Node 42 :

Char. 5: 0 → 1

Char. 8: 0 → 1

Char. 10: 0 → 1

Char. 11: 0 → 1

Char. 12: 1 → 0

Char. 26: 0 → 1

Char. 28: 0 → 1

Char. 33: 0 → 1

Char. 34: 0 → 1

Char. 44: 0 → 1

Char. 64: 0 → 1

Char. 66: 0 → 1

Char. 76: 0 → 1

Char. 77: 1 → 0

Char. 78: 1 → 0

Node 43 :

Char. 18: 1 → 0

Char. 24: 0 → 1

Char. 34: 1 → 0

Char. 43: 1 → 0

Char. 51: 0 → 1

Char. 53: 0 → 1

Char. 54: 0 → 1

Char. 58: 0 → 1

Char. 60: 0 → 1

Char. 61: 1 → 0

Char. 65: 0 → 1

Char. 71: 0 → 1

Char. 72: 0 → 1

Char. 73: 0 → 1

Char. 81: 0 → 1

Char. 84: 1 → 0

Char. 88: 1 → 0

Char. 91: 2 → 1

Node 44 :

Char. 13: 1 → 0

Char. 64: 1 → 0

Node 45 :

Char. 6: 0 → 1

Char. 37: 0 → 1

Char. 40: 1 → 0

Char. 72: 0 → 1

Char. 75: 0 → 1

Char. 82: 1 → 0

Node 46 :

Char. 0: 0 → 1

Char. 27: 0 → 1

Char. 29: 0 → 1

Char. 99: 0 → 2

Node 47 :

Char. 7: 0 → 1

Char. 34: 1 → 0

Char. 36: 0 → 1

Char. 63: 1 → 0

Char. 64: 1 → 0

Char. 65: 0 → 1

Char. 66: 1 → 0

Char. 75: 1 → 0

Char. 76: 1 → 0

Char. 82: 0 → 1

Char. 84: 1 → 0

Char. 99: 2 → 1

Char. 100: 2 → 0

Node 48 :

Char. 93: 0 → 1

Node 49 :

Char. 95: 0 → 1

Char. 97: 0 → 1

Char. 98: 0 → 1

Node 50 :

Char. 95: 1 → 2

Node 51 :

Char. 94: 0 → 1

Table S1. Specimens added to phylogenetic analysis.

| **Species** | **Specimen personally observed** | **Citation (if applicable)** |
| --- | --- | --- |
| †*Gasterorhamposus zuppichini*  †*Gerpegezhus paviai*  †*Paraeoliscus robinetae*  †*Aeoliscus heinrichsi*  †*Parasynarcualis longirostris*  †*Urosphen fistularis*  †*Aulostomoides tyleri*  †*Eekaulostomus cuevasae*  †*Protosyngnathus sumatrensis*  †*Solenorhynchus elegans*  †*Calamostoma lesiniforme*  †*Prosolenostomus lessinii*  †*Pseudosyngnathus opisthopterus*  *Syngnathus floridae*  *Doryichthys martensii*  †*Hipposyngnathus neriticus*  †*Pshekhagnathus polypterus*  *Phyllopteryx dewysea*  †*Hippotropiscis frenki*  *Acentronura gracilissima*  *Cylix tupareomanaia*  *Hippocampus pontohi*  †*Hippocampus sarmaticus*  *Hippocampus reidi*  †*Hippocampus slovenicus* | n/a  n/a  n/a  YPM VP 001611-13  MNHN BOL 106  MNHN BOL 117  MNHN BOL 101  n/a  n/a  n/a  MNHN BOL 50  MNHN BOL 560  MNHN BOL 123  n/a  n/a  n/a  n/a  n/a  n/a  n/a  n/a  n/a  n/a  n/a  n/a | Orr (1995)  Bannikov and Carnevale (2012)  Blot (1980)  n/a  n/a  n/a  n/a  Cantalice and Alvarado-Ortega (2016)  Murray (2022)  Bannikov and Carnevale (2017)  Bannikov and Carnevale (2017)  Bannikov and Carnevale (2017)  n/a  Bergert and Wainwright (1996)  Scan in Adam et al. (2015)  Přikryl et al. (2011)  Bannikov et al. (2017)  X-Ray and Scan in Stiller et al. (2015)  Žalohar and Hitij (2012)  X-Ray and Scan in Short et al. (2021)  X-Ray and Scan in Short et al. (2021)  X-Ray and Scan in Short et al. (2018)  Žalohar et al. (2009)  Scan in Van Cauter et al. (2011)  Žalohar et al. (2009) |
|  |  |  |
